# Two case reports on the identification and management of hypervirulent *Klebsiella pneumoniae* isolated in Nebraska, USA

**DOI:** 10.1128/asmcr.00233-25

**Published:** 2026-03-11

**Authors:** John S. Stevenson, Emily D. Dyer, Paul D. Fey, Trevor C. Van Schooneveld, Michael R. Wiley, Peter C. Iwen, Ulrike Carlino-MacDonald, Thomas A. Russo

**Affiliations:** 1 Department of Pathology, Microbiology, and Immunology, University of Nebraska Medical Center 12284, Omaha, Nebraska, USA; 2 Division of Infectious Disease, Department of Internal Medicine, University of Nebraska Medical Center 12284, Omaha, Nebraska, USA; 3 Nebraska Public Health Laboratory 220788, Omaha, Nebraska, USA; 4 Veterans Administration Western New York Healthcare System, Buffalo, New York, USA; 5 Department of Medicine, Jacobs School of Medicine and Biomedical Sciences, University at Buffalo 12292 , Buffalo, New York, USA; Rush University Medical Center, Chicago, Illinois, USA

**Keywords:** clinical microbiology, hypervirulent *Klebsiella pneumoniae*, emerging pathogen, molecular diagnostics

## Abstract

**Background:**

Hypervirulent *Klebsiella pneumoniae* (hvKp) is a globally emerging pathotype known for causing severe and disseminated infections across multiple organ systems. Diabetes is a risk factor for hvKp infection; however, otherwise healthy individuals from the community are also at risk.

**Case Summary:**

Two patients from Nebraska presented with complex abscesses of unknown origin. Both cases required interventional procedures and prolonged hospitalization. PCR-based testing, animal studies, and whole-genome sequencing analysis confirmed that hvKp was the offending pathotype.

**Conclusion:**

This report describes the first confirmed cases of hvKp infection in Nebraska, USA. These cases demonstrated the utility of using molecular methods to identify hvKp. The microbiology lab can be operationalized to provide valuable insights into the genetic makeup of pathogens, inform the institutional clinical decision-making processes, and epidemiological surveillance. This study highlights the collaborative relationships between the Infectious Diseases staff and the Clinical Microbiology Laboratory to work up complex cases.

## INTRODUCTION

*Klebsiella pneumoniae* is commonly found in the human gastrointestinal tract and can act as an opportunistic nosocomial pathogen, causing a range of infections such as pneumonia, wounds, and infections of the urinary tract and bloodstream (1). Hypervirulent *Klebsiella pneumoniae* (hvKp) is a distinct pathotype capable of causing invasive and disseminated infections, most notably liver infections (2, 3). First described in 1986 in Taipei, Taiwan, in a patient with *K. pneumonia* liver abscess associated with septic endophthalmitis (4), hvKp has now been identified in all six World Health Organization (WHO) regions (2, 3, 5). Diabetes is a risk factor; however, otherwise healthy individuals from the community are also at risk (2). Identification of hvKp is clinically important because it can guide appropriate treatment strategies. Key examples include the need for thorough evaluation and source control of occult abscesses (6, 7), awareness and immediate recognition of endophthalmitis (4, 8, 9), and the recommendation to use the largest bore catheter possible during percutaneous abscess drainage to minimize clogging (10). Furthermore, one study highlighted the importance of improved clinical management and surveillance of hvKp infections (11). In this analysis, patients with hvKp infections experienced more severe disease and higher 14- day mortality compared to those with non-hypervirulent strains.

## CASE PRESENTATION

### Case 1

**Fig 1:**
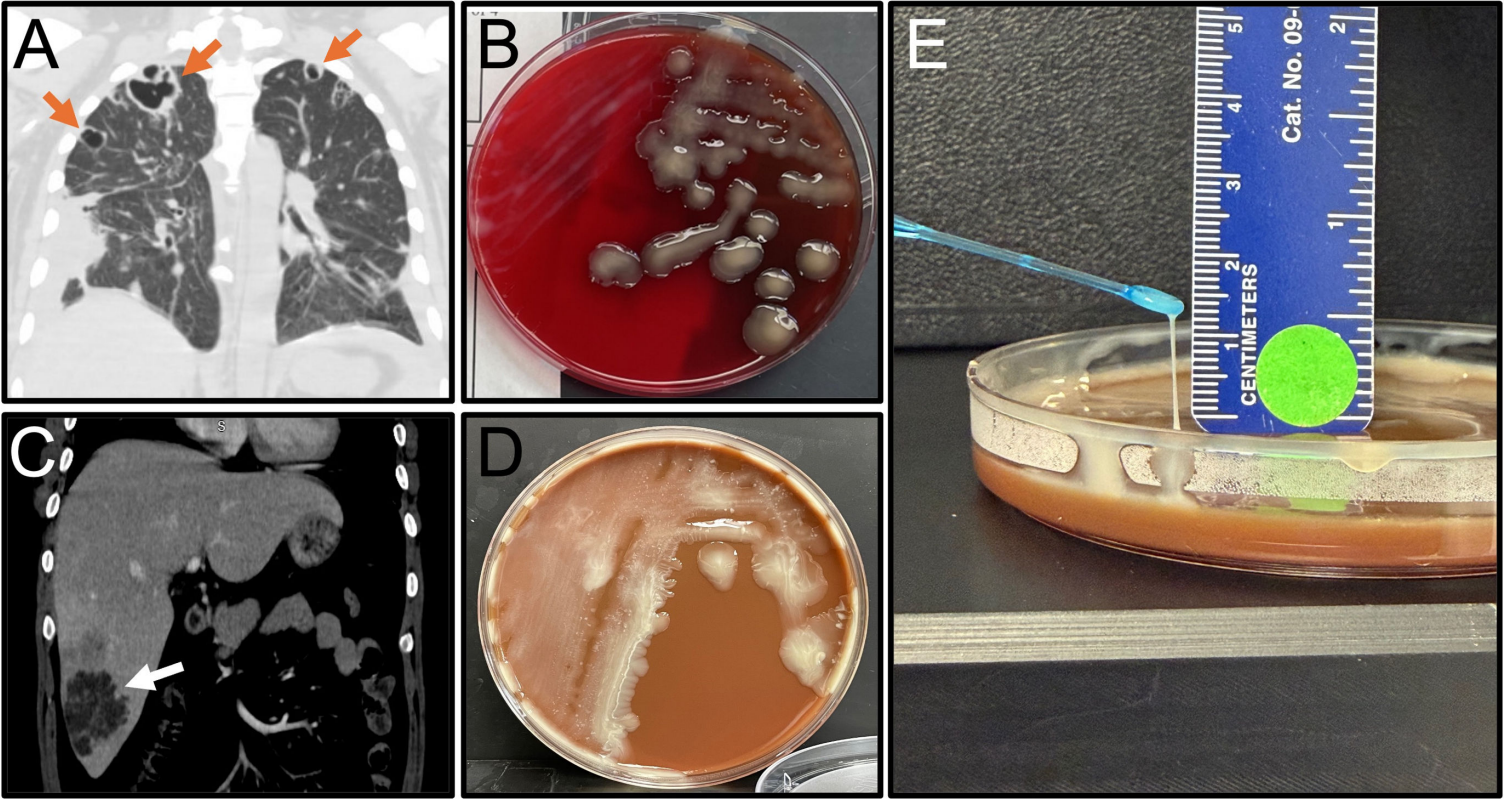
Clinical imaging and phenotypic morphologies of hypervirulent *K. pneumoniae*. Case 1: (**A**) imaging of bilateral cavitary lung lesions as dark spots (indicated with orange arrows) on the upper right and left lobes, and (**B**) representative colony morphology on sheep blood agar from the prostate abscess culture. Case 2: (**C**) computed tomography of liver with abscess (indicated with white arrow), and (**D**) representative colony morphology on chocolate agar from the liver abscess culture. (**E**) Representative string test of *K. pneumoniae* isolated from Case 2 grown on chocolate agar showing >5 mm length.

A middle-aged man with poorly controlled diabetes and prior residence in Mexico was transferred to our facility for further evaluation with possible surgical intervention due to complications from *K. pneumoniae* bacteremia. Percutaneous drainage of liver abscesses had been performed at the external facility, with cultures positive for *K. pneumoniae* (isolates unavailable). Imaging revealed bilateral cavitary lung lesions with loculated effusions (Fig. 1A), multiple liver abscesses, bilateral renal abscesses, and prostatic abscesses. MRI of his brain showed numerous scattered punctate foci in the cerebellar hemispheres suggestive of septic emboli, while clinical exam revealed endogenous endophthalmitis. Upon transfer to our facility, one admission blood culture was collected, after which the patient was started on ceftriaxone (2 g IV BID). The single admission blood culture showed no growth after 5 days of incubation using an automated blood culture system (BD BACTEC, Sparks, MD, USA). Culture from the drainage of the prostatic abscess yielded rare *K. pneumoniae*, identified by MALDI-TOF Bruker Biotyper Claim 6 database (Bruker, Billerica, MA, USA). The organism demonstrated a hypermucoviscous colony phenotype (Fig. 1B), supporting a clinical suspicion for the hvKp pathotype. Phenotypic antimicrobial susceptibility testing (AST) (MicroScan MIC Panel NM56, Beckman Coulter, Inc., West Sacramento, CA, USA) showed resistance to only ampicillin (MIC >16 µg/mL, panel range 8–16 μg/mL). The patient was treated with ceftriaxone (2 g IV BID) until hospital day 14, when he was discharged on oral levofloxacin (750 mg daily) with a planned treatment duration of at least 1–2 months based on radiographic resolution of abscesses. Approximately 2 months after discharge, he was readmitted with septic shock and respiratory failure due to multifocal pneumonia after receiving a course of corticosteroids for endophthalmitis. Imaging during this admission indicated improvement in his abscesses. Two sets of admission blood cultures were taken and were negative after 5 days of incubation using an automated blood culture system (BD BACTEC, Sparks, MD, USA). Also, upon admission, a rapid molecular pneumonia panel (BioFire, Salt Lake City, UT, USA) detected >=10,000,000 copies/mL of *K. pneumoniae* group without resistance genes from a sputum specimen. Sputum cultures grew a hypermucoviscous *K. pneumoniae* isolate, again suggesting the hvKp pathotype. Phenotypic AST now showed resistance to ampicillin (MIC >16 µg/mL, panel range 8–16 μg/mL), levofloxacin (MIC 4 μg/mL, panel range 0.5–4 μg/mL), with intermediate susceptibility to tetracycline (MIC 8 g/mL, panel range 4–8 μg/mL), minocycline (MIC 8 g/mL, panel range 4–8 μg/mL), and cefuroxime (MIC 16 g/mL, panel range 4–16 μg/mL). During hospitalization, he underwent right-eye vitrectomy, with the culture being negative. After 4 days of initial broad-spectrum therapy (vancomycin 1 g IV daily with dosing modifications based on renal function and levels and piperacillin-tazobactam 4.5 g IV q8h), treatment was de-escalated to cefazolin (2 g IV q8h) for 9 days and then trimethoprim-sulfamethoxazole (800–160 mg BID). On hospital day 27, he was discharged to a long-term care facility (LTAC) with treatment (trimethoprim-sulfamethoxazole 800–160 mg BID), duration to be guided by radiographic resolution of abscesses. After discharge from the LTAC to home, the patient discontinued treatment (trimethoprim-sulfamethoxazole 800–160 mg BID) on his own after completing an additional month of therapy (total: 2 months of therapy). At a follow-up visit ~3 months after his second admission, he was found to be doing well without antibiotics.

### Case 2

A middle-aged male originally from Southeast Asia presented with 3 weeks of worsening abdominal pain. Initial evaluation was remarkable for elevated aspartate aminotransferase (AST 78 U/L; reference 15–41 U/L) and alanine aminotransferase (ALT 147 U/L; reference 7–52 U/L). Computed tomography (CT) of the abdomen showed an indeterminate cystic lesion in the right hepatic lobe and a mesenteric fluid collection (Fig. 1C). Two admission blood culture sets were obtained before empiric antibiotic therapy with ceftriaxone (2 g IV BID) and metronidazole (500 mg TID) was initiated. Approximately 2 days after admission, percutaneous drainage of the hepatic abscesses yielded purulent fluid for culture. All four admission blood cultures became positive within ~11 h (BD BACTEC, Sparks, MD, USA). A rapid molecular blood identification panel (BCID2, BioFire, Salt Lake City, UT, USA) detected *K. pneumoniae* group without resistance genes. Admission blood cultures recovered a pure isolate of *K. pneumoniae*, identified by MALDI-TOF Bruker Biotyper Claim 6 database (Bruker, Billerica, MA, USA) exhibiting a hypermucoviscous phenotype (Fig. 1D). The AST results (MicroScan MIC Panel NM56, Beckman Coulter, Inc., West Sacramento, CA, USA) showed resistance to only ampicillin (MIC >16 µg/mL, panel range 8–16 μg/mL). The hepatic abscess culture grew moderate amounts of hypermucoviscous *K. pneumoniae,* and the AST results matched the blood culture results. Subsequent blood cultures, two sets each collected at 9 and 11 days after admission, were negative after 5 days of incubation. At 9 days post-admission, the patient developed severe left knee pain with concern for metastatic infection, but investigation revealed acute gout. After 14 days of admission, the metronidazole (500 mg TID) was stopped, and he was discharged on ceftriaxone (2 g IV daily) for 3 weeks. He was seen 3 weeks after discharge, at which time he was transitioned to oral levofloxacin (750 mg daily). He was seen again ~1 month later, at which time antibiotics were discontinued due to decreased size of liver abscess on CT imaging.

## DISCUSSION

Clinical suspicion for hvKp was high for both cases, but laboratory diagnostics at UNMC were limited to the string test (Fig. 1E). The string test, although commonly employed to identify hvKp, lacks optimal sensitivity and specificity, and false negatives are particularly problematic (12–14). More recently, PCR-based detection of the five plasmid-linked virulence genes (*iucA, iroB, peg344, rmpA*, and *rmpA2*) has emerged as a promising laboratory-developed test to distinguish hvKp from “classical” *K. pneumoniae* (cKp) (13, 15, 16). Therefore, ~1 month after isolation of the bacteria, we reached out to T.A.R. to perform PCR-based studies, as described in a previous study (13) (specific primers used and PCR conditions described in that reference’s Supplemental Table 1), for Case 1 prostate isolate and Case 2 hepatic and blood isolates. All three isolates were PCR positive for all five hvKp biomarkers (*iucA, iroB, peg344, rmpA*, and *rmpA2*). While imperfect, this remains one of the most practical molecular methods for hvKp identification (13, 15, 16). Furthermore, using a gold-standard murine sepsis model (not a routine laboratory method) in accordance with IRBNet#1580121 at Veterans Administration Western New York Healthcare System, outbred male CD1 mice were challenged subcutaneously with the case isolates in accordance with methods described in (13, 15). These studies confirmed their hvKp pathotype (Case 1 prostate abscess, Kp101724, LD_50_ 1.88 × 10³ CFU, *n* = 5; Case 2, liver abscess, Kp101924, LD_50_ 2.43 × 10³ CFU, *n* = 5) in contrast to an LD_50_ >1.0 × 10^7^ CFU seen with the cKp pathotype.

Whole-genome sequencing (WGS) of both the chromosome and plasmids has been used to study hvKp pathogenesis, evolution, and global spread of the dominant sequence type (ST) 23 (17–19). Plasmid-level genomics analysis to calculate Jaccard and Mash distances of hvKp plasmids in comparison with the ancestor pLVPK was the second-most accurate characteristic for predicting hvKp (15, 16). Approximately 1 month after initial isolation, WGS analysis (Oxford Nanopore Technologies (ONT) ElysION automated sequencer, Oxford, UK) with BugSeq Bioinformatic pipeline (20) was performed to assess genetic relatedness, given concern for clonal spread at the hospital. WGS analysis from Case 1 documented that this isolate encoded a KL1 capsular locus and was ST23. In contrast, the Case 2 isolate encoded a KL2 capsular locus and was ST65. The assembled plasmid sequences confirmed the isolates harbored hvKp-specific virulence plasmids containing the same loci detected by PCR, and their Jaccard and Mash distances to pLVPK were consistent with hvKp distributions reported previously (15, 16, 18, 20). Comparative genomics excluded clonal spread and horizontal gene transfer within the healthcare setting, indicating independent acquisition of hvKp strains in two unrelated patients without recent travel history, suggesting earlier acquisition and prolonged colonization. Therefore, if suspicion arises for specific pathotypes of concern, such as hvKp, seeking help from experts in the field (as was done in this study), assistance from the Association of Public Health Laboratories, commercial labs, or other entities is recommended.

### Conclusion

This report describes the first confirmed cases of hvKp infection in Nebraska, USA. These cases demonstrated the utility of using molecular methods to identify hvKp. The microbiology lab can be operationalized to provide valuable insights into the genetic makeup of pathogens, inform the institutional clinical decision-making processes, and epidemiological surveillance. This study highlights the collaborative relationships between the Infectious Diseases staff and the Clinical Microbiology Laboratory to work up complex cases.

## Data Availability

WGS data are available in NCBI under accession no. SAMN50444771 for Case 1 and SAMN50444770 for Case 2.
